# Metagenome-Assembled Genome Sequence of *Phormidium* sp. Strain SL48-SHIP, Isolated from the Microbial Mat of Salt Lake Number 48 (Novosibirsk Region, Russia)

**DOI:** 10.1128/MRA.00651-19

**Published:** 2019-08-01

**Authors:** Aleksey S. Rozanov, Aleksandra A. Shipova, Alla V. Bryanskaya, Sergey E. Peltek

**Affiliations:** a Federal Research Center Institute of Cytology and Genetics of the Siberian Branch of the RAS, Novosibirsk, Russia; Loyola University Chicago

## Abstract

The Phormidium sp. strain SL48-SHIP genome was obtained from metagenomics sequencing of the microbial mat of Salt Lake No. 48 (54.201806 N, 78.179194 E; Novosibirsk Region, Russia). The sequenced and annotated genome is 4,384,607 bp and encodes 3,807 genes.

## ANNOUNCEMENT

Bacteria of the genus *Phormidium* are blue-green filamentous algae (Cyanobacteria) belonging to the order Oscillatoriales. They produce various anatoxins that pose a risk to animal health (1). Some genomes of the genus *Phormidium* in other regions of the planet were previously sequenced (2). One of the many salt lakes of the Novosibirsk Region is Salt Lake No. 48 (3). Recently, the genome of Halorubrum sp. strain 48-1-W was sequenced from this lake (4).

A sample of the microbial mat was taken from Salt Lake No. 48 (54.201806 N, 78.179194 E; Novosibirsk Region, Russia). The sample was taken in sterile Falcon tubes, and gloves were used. The sample was stored in alcohol at −70°C. Total DNA was isolated using genomic DNA from a soil kit (NucleoSpin soil) and using the default protocol. Libraries for metagenome sequencing (with an average length of 600 bp) were prepared at the Center of Genomic Studies, Siberian Branch of the Russian Academy of Sciences (ICG SB RAS), with a NEBNext Ultra DNA library prep kit for Illumina. Metagenome sequencing (paired end) was performed on a NovaSeq platform (Illumina) at Genetico LLC using an NovaSeq 6000 S2 reagent kit (200 cycles).

A total of 398,852,702 100-bp reads were sequenced. The reads were trimmed with Trimmomatic version 0.36 (using the options MINLEN:95 and CROP:97) (5). *De novo* assembly of short reads into scaffolds was performed using SPAdes version 3.11.1 using default parameters (with the option –only-assembler) (6). Contigs shorter than 1,000 bp were deleted.

Binning of metagenomic scaffolds into discrete clusters (in which one bin represents one genome) was carried out using MaxBin version 2.2.4 with default parameters (7). Then, we described one of the resulting genomes, with a coverage of 65.31×. We compared this genome with the reference genomes of the most closely related cyanobacteria using an average nucleotide identity (ANI) calculator (http://enve-omics.ce.gatech.edu/ani/) with default parameters. The genomes of *Phormidium* sp. strain HE10JO (identity, 93.37%), Phormidium willei BDU 130791 (identity, 90.97%), and *Phormidium* sp. strain OSCR (identity, 88.17%) were closest to our genome. Thus, this genome was assigned to *Phormidium* sp. strain SL48-SHIP.

A total of 537 contigs yielded a genome sequence 4,384,607 bp long; the GC content is 52.00%, and the *N*_50_ is 13,686 (evaluated using QUAST with default parameters [8]). Open reading frame (ORF) prediction and automatic annotation were performed using the NCBI Prokaryotic Genome Annotation Pipeline (PGAP) with the default parameters (9). The complete genome sequence contains 3,807 genes, 3,760 coding sequences (CDS), rRNAs (1 5S rRNA and 2 23S rRNAs), 41 tRNAs, and 3 noncoding RNAs (ncRNAs).

### Data availability.

The raw metagenomics data have been deposited at DDBJ/EMBL/GenBank under the accession no. SRR7943696. The draft genome sequence for *Phormidium* sp. strain SL48-SHIP has been deposited at DDBJ/EMBL/GenBank under the accession no. SIHH00000000. The 537 contigs have been deposited under accession no. SIHH01000001 to SIHH01000537.
